# Coil embolization of a giant pseudoaneurysm associated with a disrupted axillary artery: a case report

**DOI:** 10.1186/s42155-023-00408-4

**Published:** 2024-03-11

**Authors:** Naoki Oka, Akira Kuriyama, Yukio Ishisaka

**Affiliations:** 1 Emergency and Critical Care Center, Kurashiki Central Hospital, 1-1-1 Miwa Kurashiki, Okayama, 710-8602 Japan; 2 Department of Diagnostic Radiology, Kurashiki Central Hospital, Okayama, Japan

**Keywords:** Pseudoaneurysm, Axillary artery, Embolization, Therapeutic

## Abstract

**Background:**

Stent-graft placement is generally used to treat pseudoaneurysm (PSA) of the axillary artery (AA) trunk to maintain the patency of peripheral vessels. Coil embolization of a PSA associated with a disrupted AA trunk has rarely been reported.

**Case presentation:**

A 54-year-old woman presented with swelling of her right shoulder. She had had a right proximal humeral fracture 12 years earlier. Contrast-enhanced computed tomography (CECT) and subsequent angiograms revealed a giant PSA at the disrupted, distal right AA. There were collateral flows to the brachial artery from the proximal to the right AA. To preserve collateral flows to the brachial artery, selective embolization of the inflow artery that derived from the distal AA was performed with hydrogel-coated coils. The post-embolization arteriogram showed no flow into the PSA, but collateral flows to the brachial artery we preserved. The post-embolization course was uneventful. The patient regained warmth in her right arm and hand on post-embolization day 4. Repeat CECT on post-embolization day 9 confirmed blood-flow to her right radial artery.

**Conclusions:**

While a stent-graft should be used if the AA trunk can be preserved, coil embolization should be considered for PSA if the AA trunk is disrupted but collaterals are preserved.

## Background

The axillary artery (AA) is the main vessel responsible for arterial supply of the upper limb. It commences from the subclavian artery at the outer border of the first rib and terminates at the outer border of the teres major muscle, where it transitions to the brachial artery. The AA is anatomically close to the glenohumeral joint and proximal humerus.

Pseudoaneurysms (PSAs) of the AA are rare [1]. They are associated with blunt trauma (such as proximal humeral fractures [2] and anterior glenohumeral dislocation [3]), penetrating injuries, and iatrogenic trauma (such as pacemaker placement [4]). Endovascular treatment is preferred over surgery because of technical feasibility related to the anatomical nature of the AA, as well as improved morbidity and mortality [5–7]. Stent-graft placement is used for the treatment of PSA in the AA main trunk to preserve the patency of peripheral vessel [8]. However, we used coil embolization to treat a case of PSA associated with the main trunk of AA disrupted by a humeral fracture.

## Case presentation

**Fig. 1:**
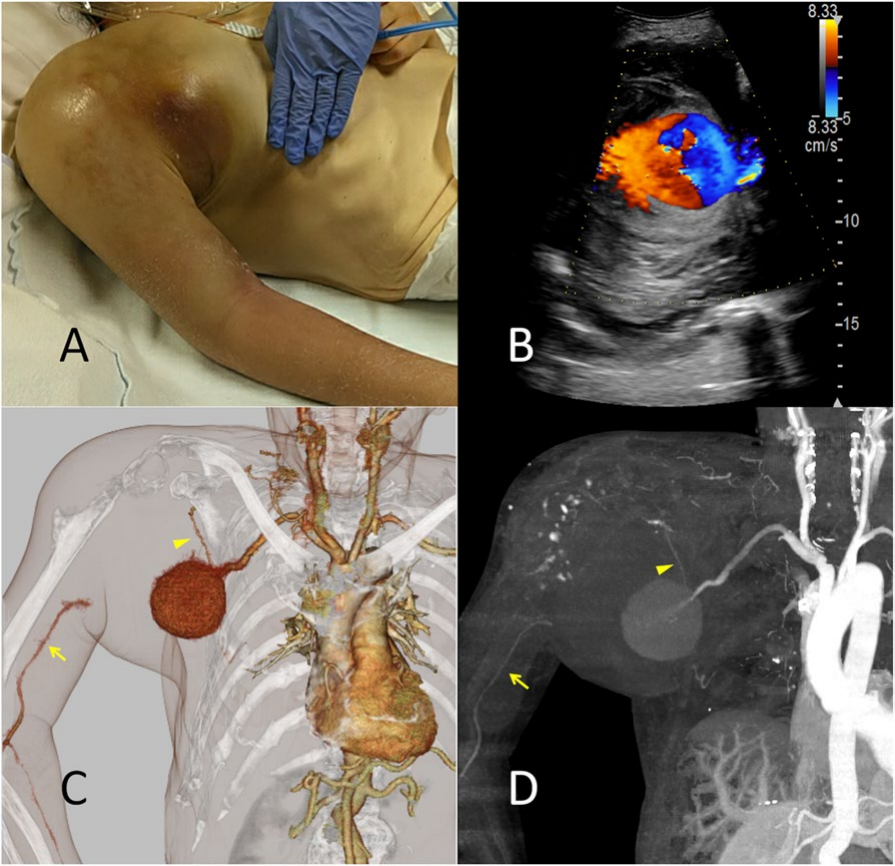
Photograph of the right swollen shoulder of the patient (**A**). The ‘Yin-Yang” sign detected via Doppler ultrasonography (**B**). The volume rendering image (**C**) and maximum intensity projection (**D**) of the pseudoaneurysm of the disrupted, right axillary artery and collateral blood-flow (arrowhead) to the right brachial artery (arrow) obtained via three-dimensional computed tomography angiography

A 54-year-old woman with schizophrenia presented with right shoulder swelling. She had had a right proximal humeral fracture 12 years earlier. Surgery was indicated for the fracture, but the family opted for conservative treatment because of concurrent Takotsubo cardiomyopathy. She had been unable to move her right arm spontaneously thereafter. During her admission at another hospital for rehabilitation a month before the referral to us, her right shoulder developed a swelling, which deteriorated over 3 weeks. She was then referred to our hospital. On admission, her right shoulder was markedly swollen (Fig. 1A) and painful on passive motion and had a purple skin color. Her right arm was cold. Doppler ultrasonography showed a “Yin-Yang sign” in the distal AA (Fig. 1B), which indicated a partial thrombosis of the PSA [9], while blood-flow was not detected in her right radial artery.

Ultrasonography around the right humeral articulation revealed a 6-cm cystic structure located 5 cm from the skin, which was supplied by an artery. Contrast-enhanced computed tomography (CECT) revealed a right proximal humeral fracture with posterior dislocation of the right humeral articulation and a 6-cm-diameter PSA connected to the right AA trunk, which were separated by a large hematoma (Fig. 1C and D).

**Fig. 2:**
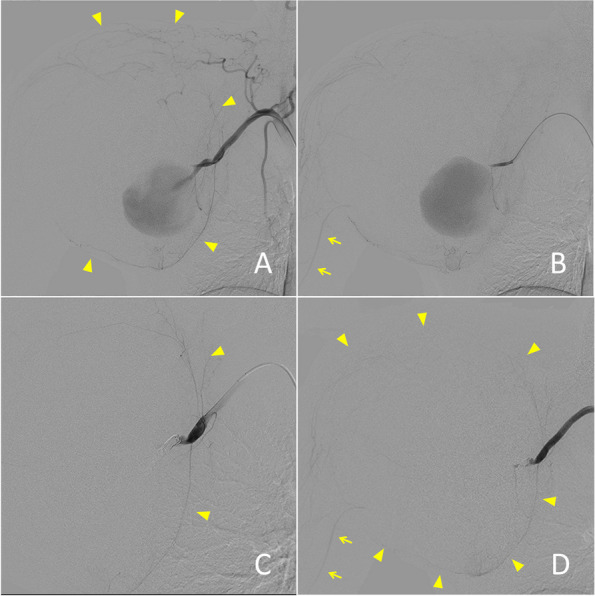
Digital subtraction angiography (DSA) of the right axillary artery confirmed a giant pseudoaneurysm at the disrupted, distal right axillary artery and near the origin of collateral flows (arrowheads) (**A**) and blood-flow to the brachial artery (arrows) (**B**). DSA showed embolization of the inflow artery to the pseudoaneurysm with coils (**C**) and preservation of the collateral flows (arrowheads) to the brachial artery (arrows) (**D**)

Angioplasty of the AA was deemed impossible after consultation with the vascular and orthopedic surgeons. We decided to perform coil embolization to reduce the risk of extensive bleeding during the probable amputation of the arm. We placed a 4-Fr short sheath (Terumo Medical, Shibuya, Japan) in the right femoral artery and a Goodtec catheter JB2 (Medikit, Tokyo, Japan) in the brachiocephalic artery. Right AA angiography confirmed a giant PSA at the distal right AA (Fig. 2A). The right AA trunk was thought to be disrupted, given the lack of a vessel beyond the PSA. We identified collateral flows to the brachial artery from the proximal to the right AA (Fig. 2B). To preserve collateral flows to the forearm, selective catheterization of the inflow artery (diameter of 5 mm and length of 20 mm) to the PSA, which derived from the distal AA, was directly performed using Goodtec catheter JB2 (Fig. 2C). With the edge of the coil placed slightly out from the microcatheter (LIGHTHOUSE 0.021-inch microcatheter with an outer diameter of 2 Fr at the tip [PIOLAX, Yokohama, Japan]) into the PSA, we pushed the microcatheter back and forth to stack four coils (AZUR CX, Terumo Medical: 5 mm × 16 cm, 4 mm × 13 cm, and two 3 mm × 8 cm coils) at the inflow artery. The arteriogram showed no flow into the PSA, but collateral flows into the brachial artery were preserved (Fig. 2D).

**Fig. 3:**
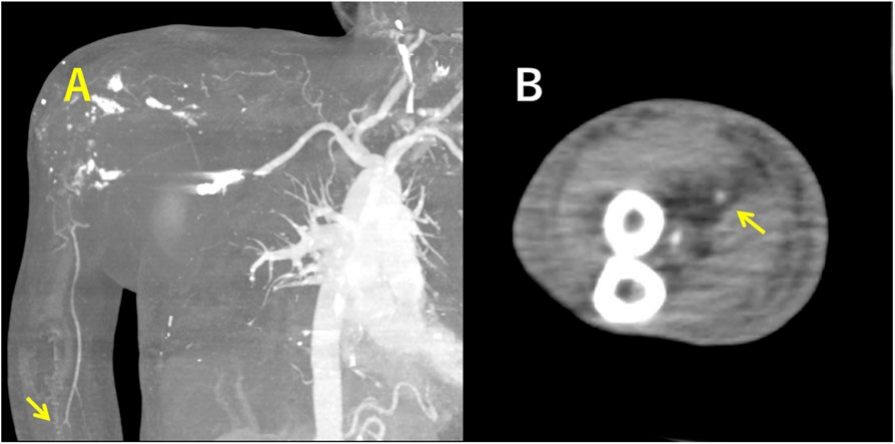
Presence of blood-flow at the right radial artery (arrow) on the maximum intensity projection of computed tomography angiography (**A**) and on the axial view of contrast-enhanced computed tomography (**B**)

The post-procedural course was uneventful. Based on the treatment for thromboangiitis obliterans, the patient was administered aspirin 100 mg and intravenous alprostadil 10 μg daily. She regained warmth in her right arm and hand, and her sense of touch in the hand improved on post-embolization day 4. Repeat CECT on post-embolization day 9 confirmed blood-flow to her right radial artery (Fig. 3). Thrombus formation within and no inflow into the PSA were also confirmed. Therefore, aspirin and alprostadil were discontinued. She was subsequently transferred to another hospital for rehabilitation. At the one-year follow-up consultation with her primary care physician, the swelling of her right shoulder had resolved; blood-flow to her radial artery was confirmed via ultrasonography. Thus, angiography was not repeated.

## Discussion

Treatments for AA-PSA reported in previous case reports and series include surgery, ultrasound-guided compression [10], percutaneous thrombin injection [11], and endovascular treatment (stent-graft placement and embolization) [8]. We opted for embolization over surgery owing to the high risk of intraoperative bleeding and anastomotic leak, as well as chronic nerve compression. Surgery is indicated if a hematoma compresses a nerve. Symptomatic nerve compression for ≥ 3.5 days leads to severe nerve damage [12]. Furthermore, surgery can be technically demanding because of the anatomical nature of AA and result in significant morbidity and mortality [5–7]. In our patient, the nerve may have been compressed for ≥ 3 weeks; thus, nerve damage, if present, could persist even after surgery [12]. Generally, it is easier to approach a vessel with coil embolization than with stent-graft placement, with a lower risk of distal embolism. In our patient, stent-graft placement was inappropriate because CECT did not show the patency of the distal AA. The angiogram showed blood-flow to the right forearm via collateral flows from the AA. Coil embolization of the AA trunk was considered possible because blood-flow to the forearm was preserved.

To maintain the position of a coil within an inflow vessel, interventional radiologists use a balloon to decrease the blood-flow [13] or temporarily detain part of the coil as an anchor in a target vessel branch. In our patient, we created a scaffold by using coils with a strong radial force and densely packed them to embolize a short segment of the right AA, ensuring that collateral flows to the forearm were not excluded. We chose hydrogel-coated coils, which expand in volume within the target vessel, thereby enhancing its obstruction effect [14].

## Conclusion

While a stent-graft should be used if the AA trunk can be preserved, coil embolization is a treatment option for PSA if the AA trunk is disrupted but collaterals are preserved.

## Data Availability

Not applicable.
